# Myocardial Viability, Functional Status, and Collaterals of Patients With Chronically Occluded Coronary Arteries

**DOI:** 10.3389/fcvm.2021.754826

**Published:** 2021-11-12

**Authors:** Xueyao Yang, Jinfan Tian, Lijun Zhang, Wei Dong, Hongzhi Mi, Jianan Li, Jiahui Li, Ye Han, Huijuan Zuo, Jing An, Yi He, Xiantao Song

**Affiliations:** ^1^Department of Cardiology, Beijing Anzhen Hospital, Capital Medical University, Beijing, China; ^2^Department of Radiology, Beijing Anzhen Hospital, Capital Medical University, Beijing, China; ^3^Department of Nuclear Medicine, Beijing Anzhen Hospital, Capital Medical University, Beijing, China; ^4^Department of Radiology, Beijing Friendship Hospital, Capital Medical University, Beijing, China; ^5^Department of Community Health Research, Beijing Institute of Heart, Lung and Blood Vessel Disease, Beijing Anzhen Hospital, Capital Medical University, Beijing, China; ^6^Siemens Shenzhen Magnetic Resonance Ltd., Shenzhen, China

**Keywords:** chronic total occlusion, myocardial viability, coronary artery disease, cardiovascular magnetic resonance, cardiac function

## Abstract

**Objective:** Viability and functional assessments are recommended for indication and intervention for chronic coronary total occlusion (CTO). We aimed to evaluate myocardial viability and left ventricular (LV) functional status by using cardiovascular magnetic resonance (CMR) and to investigate the relationship between them and collaterals in patients with CTO.

**Materials and Methods:** We enrolled 194 patients with one CTO artery as detected by coronary angiography. Patients were scheduled for CMR within 1 week after coronary angiography.

**Results:** A total of 128 CTO territories (66%) showed scar based on late gadolinium enhancement (LGE) imaging. There were 1,112 segments in CTO territory, while only 198 segments (18%) subtended by the CTO artery showed transmural scar (i.e., >50% extent on LGE). Patients with viable myocardium had higher LV ejection fraction (LVEF) (56.7 ± 13.5% vs. 48.3 ± 15.4%, *p* < 0.001) than those with transmural scar. Angiographically, well-developed collaterals were found in 164 patients (85%). There was no significant correlation between collaterals and the presence of myocardial scar (*p* = 0.680) or between collaterals and LVEF (*p* = 0.191). Nevertheless, more segments with transmural scar were observed in patients with poorly-developed collaterals than in those with well-developed collaterals (25 vs. 17%, *p* = 0.010).

**Conclusion:** Myocardial infarction detected by CMR is widespread among patients with CTO, yet only a bit of transmural myocardial scar was observed within CTO territory. Limited number of segments with transmural scar is associated with preserved LV function. Well-developed collaterals are not related to the prevalence of myocardial scar or systolic functioning, but could be related to reduce number of non-viable segments subtended by the CTO artery.

## Introduction

Coronary chronic total occlusions (CTOs) are detected in ~15–25% of patients who undergo coronary angiography (1–4). Beneficial effects of CTO revascularization include angina relief, decreased ischemia, and improved functional status (5–7). However, while better outcomes have been shown in non-randomized studies (5–7), evidence from randomized trials suggests that CTO percutaneous coronary intervention (PCI) treatment is not superior to conservative treatment with regard to functional status and long-term outcomes (8–10). Thus, an appropriate indication of CTO intervention is crucial when considering potential benefits, challenges, and risks. Therefore, baseline characteristics of patients with CTO need to be recorded in detail. Current guidelines recommend evaluation of symptoms and ischemia burden, but myocardial viability is also recommended as it is a newly recognized potential predictor of functional recovery following successful CTO PCI (11). In this study, we assessed the myocardial viability and functional status in CTO territories by using cardiovascular magnetic resonance (CMR) imaging and investigated the relationship between them and collaterals.

## Materials and Methods

### Patients

**Figure 1:**
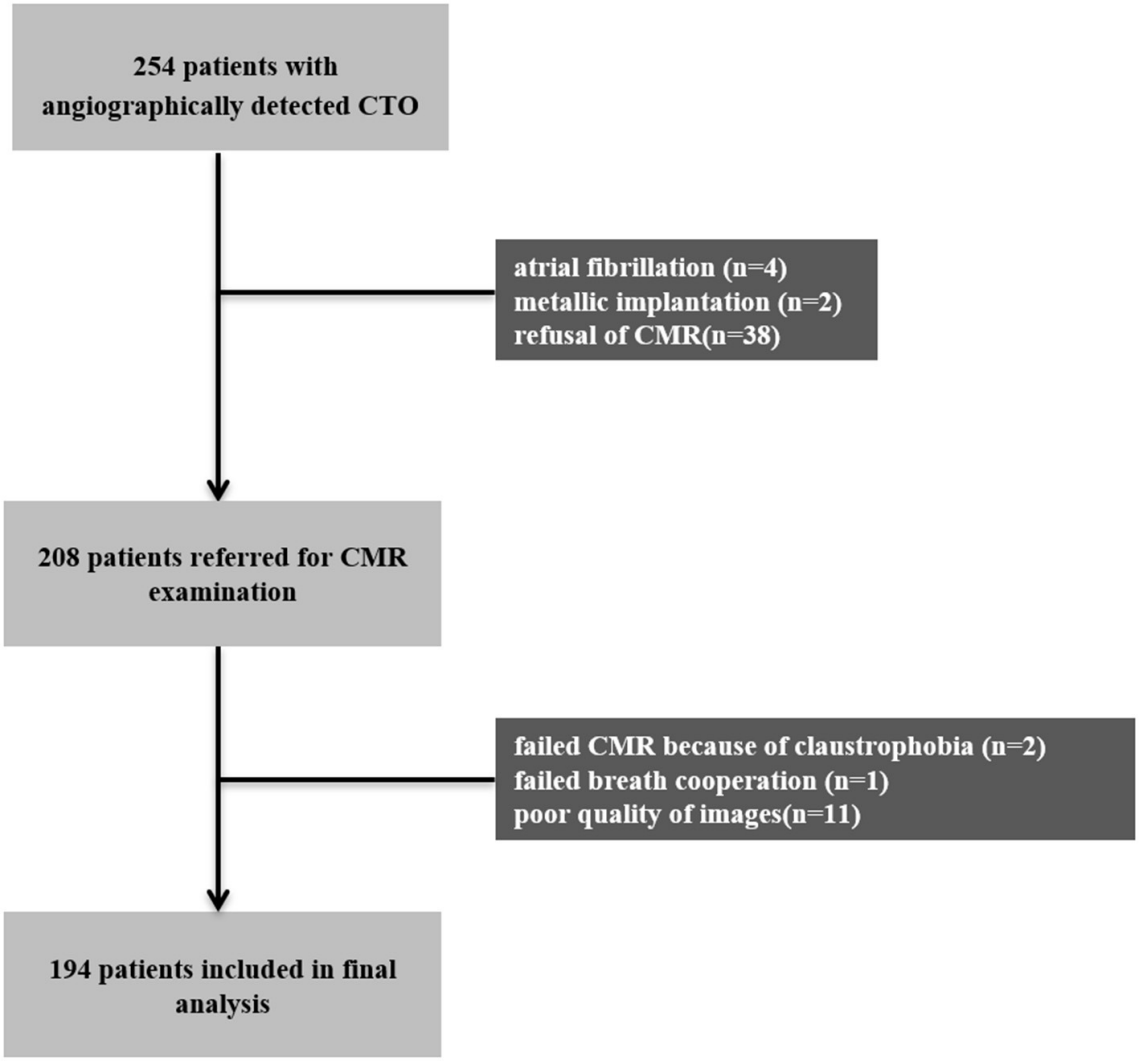
Patient and imaging flowchart. CMR, cardiovascular magnetic resonance; CTO, chronic total occlusion.

A total of 254 patients who underwent coronary angiography due to suspected angina or ischemic evidence between December 2014 and March 2020 were verified to have CTO in only one major epicardial coronary artery and were considered for inclusion in this study. The study protocol was approved by the Ethics Board of Beijing Anzhen Hospital, Capital Medical University. Patients with acute myocardial infarction within 3 months, patients with coagulation disorders, and patients who refused or were unable to undergo CMR (*n* = 46) were excluded from the study. Patients were scheduled for CMR within 1 week after coronary angiography. A total of 14 patients with poor CMR images or failed procedures were also excluded, leaving a final total of 194 eligible patients that were enrolled in the study. Figure 1 shows a schematic diagram describing the study cohort. All the patients were treated with optimal medical therapy (OMT) (medical therapy formulated by clinicians in full consideration of risk factor modification and permanent lifestyle changes) (12), regardless if the revascularization procedure succeeded or not. Written informed consent was obtained from all the patients and the study protocol conformed to the ethical guidelines of the 1975 Declaration of Helsinki.

### Coronary Angiography and Collateral Assessment

Coronary angiography was performed by using the standard method. CTO was defined as 100% stenosis with the thrombolysis in myocardial infarction (TIMI) grade 0 flow in a major epicardial coronary artery (≥2.5 mm) for at least 3 months (13). Significant non-CTO coronary disease was diagnosed if there was ≥70% lumen stenosis in a major epicardial coronary artery (≥2.5 mm), except for the left main (LM) artery or ≥50% lumen stenosis in the LM artery. The presence and state of collaterals supplying the totally occluded vessel from the contralateral artery were graded by using the Rentrop classification system (14): Grade 0 referred to no collateral circulation, grade 1 referred to collateral circulation that only supplied the occluded vessel branch, grade 2 referred to collateral circulation that partially supplied the occluded vessel trunk, and grade 3 referred to collateral circulation that completely filled the occluded vessel trunk. Patients were classified as having poorly-developed collaterals (Rentrop scores of 0 and 1) or well-developed collaterals (Rentrop scores of 2 and 3). Collateral circulation scores were independently assessed by two experienced interventional cardiologists.

### Cardiovascular Magnetic Resonance Data Acquisition and Processing

Cardiovascular magnetic resonance was performed with the Siemens 3.0-T Whole-body Scanner (MAGNETOM Verio, Tim System; Siemens Healthcare, Erlangen, Germany, UK). The CMR protocol included cine images and late gadolinium enhancement (LGE) imaging. As described in our previous study (15), 8-mm sections with no intersection gaps were obtained in the short-axis plane (from the base to the apex) and the long-axis plane of the left ventricle (LV) to perform cine cardiac MR and LGE imaging. Postprocessing analyses were performed by using the Siemens–Argus software.

Two experienced radiologists performed a visual analysis of all the images based on the American Heart Association 17-segment model of LV. Discrepancies were resolved by a discussion involving the presence of another senior investigator. Segments 1, 2, 7, 8, 13, 14, and 17 were considered left anterior descending (LAD) artery territory. Segments 5, 6, 11, 12, and 16 were considered left circumflex (LCX) artery territory. Segments 3, 4, 9, 10, and 15 were considered right coronary artery (RCA) territory (16). Taking coronary dominance into account, the inferoseptal segments, inferior segments, and inferolateral segments could be reassigned (17). The wall motion and extent of LGE were graded. The wall motion of each myocardial segment was scored on a scale of 1–4: 1 = normal, 2 = hypokinesia, 3 = akinesia, and 4 = dyskinesia. The extent of segmental wall enhancement was graded on a five-point scale: 1 = no hyperenhancement, 2 = hyperenhancement of 1–25% of tissue, 3 = hyperenhancement of 26–50% of tissue, 4 = hyperenhancement of 51–75% of tissue, and 5 = hyperenhancement of 76–100% of tissue (18, 19). LGE > 50% was considered to be a transmural scar, LGE between 1 and 50% was considered to be an endocardial scar with viable myocardium, and no LGE was considered to be absent of myocardial scar. Viable myocardium was defined as one with either no LGE or 1–50% LGE. Patients with left ventricular ejection fraction (LVEF) <50% were defined as having left ventricular dysfunction.

### Statistical Analysis

Statistical analysis was performed by using the Statistical Package for the Social Sciences (SPSS) 26.0 software (IBM Corp., Armonk, NY, USA). Normally distributed data were expressed as mean ± SD. Non-normally distributed data were expressed as median (interquartile range). Continuous variables were compared by using the *t*-test or the Mann–Whitney *U* test and categorical variables were compared by using the χ2 test or the Spearman's rank correlation test. Interobserver and intraobserver agreement were tested by using the Cohen's kappa. The correlation between wall motion score and extent of LGE was evaluated by using the Spearman's rank correlation test. A two-tailed *p* < 0.05 was considered to be statistically significant.

## Results

### Baseline Characteristics

Baseline clinical and angiographic characteristics are presented in Table 1 and are ordered according to myocardial scar formation in CTO territories. Statistical analyses demonstrated that patients with myocardial scar were likely to be males and smokers and were more likely to have hypertension. The prevalence of myocardial scar in CTO territory detected by CMR (66%) was strikingly higher than traditional clinical evidence that would suggest, as only 11% of included study patients showed pathological Q waves on ECG. Angiographically, 79 patients (41%) showed at least one concomitant main artery with severe stenosis.

**Table 1:** Baseline characteristics.

	**All patients (*n* = 194)**	**No scar in CTO territory on CMR (*n* = 66)**	**Scar detected in CTO territory by CMR (*n* = 128)**	***P***
Age	57 ± 10	56 ± 10	58 ± 10	0.067
Male	160 (82%)	46 (70%)	114 (89%)	<0.0001
Smoking	102 (53%)	25 (38%)	77 (60%)	0.004
Hypertension	115 (59%)	29 (44%)	86 (67%)	0.002
Diabetes	60 (31%)	21 (32%)	39 (30%)	0.871
Hyperlipemia	80 (41%)	28 (42%)	52 (41%)	0.878
Prior PCI	52 (27%)	11 (17%)	37 (29%)	0.079
Previous MI	45 (23%)	9 (14%)	36 (28%)	0.031
Q wave	22 (11%)	1 (2%)	21 (16%)	0.001
**Angiographic presentation**				
CTO location				
LAD	71 (37%)	31 (46%)	40 (31%)	–
LCX	21 (11%)	6 (12%)	15 (12%)	–
RCA	102 (53%)	29 (42%)	73 (57%)	–
Successful CTO revascularization	126 (65%)	49 (71%)	77 (60%)	0.058
Concomitant non-CTO lesion	79 (41%)	28 (38%)	51 (40%)	0.759
**CMR characteristics**				
Scar on CMR in CTO territory	128 (66%)	–	–	–
Wall motion abnormality in CTO territory	101 (52%)	13 (20%)	88 (75%)	<0.0001
LVEF (%)	53.3 ± 14.9	58.3 ± 12.7	50.7 ± 15.2	0.001
LVEDV (mL)	107.1 ± 44.8	90.0 ± 32.4	115.9 ± 47.8	<0.0001
LVESV (mL)	53.5 ± 37.8	38.7 ± 20.1	61.1 ± 42.4	<0.0001

*CTO, chronic total occlusion; CMR, cardiovascular magnetic resonance imaging; LAD, left anterior descending coronary artery; LCX, left circumflex coronary artery; LVEF, left ventricular ejection fraction; LVESV, left ventricular end-systolic volume; LVEDV, left ventricular end-diastolic volume; PCI, percutaneous coronary intervention; RCA, right coronary artery*.

### Myocardial Viability and Functional Status

Of the 194 patients included in this study, a total of 128 patients (66%) had myocardial scar in CTO territory detected by CMR, while 66 patients (34%) did not have myocardial scar in CTO territory detected by CMR.

Late gadolinium enhancement imaging demonstrated myocardial scar in 417 segments (37%) out of 1,112 total segments. Over 50% LGE was observed in 198 (18%) CTO-related segments, while no scar was observed in 695 (63%) CTO-related segments (see Table 2). A total of 780 segments (70%) in CTO territory showed normal wall motion (score = 1). The Spearman's rank correlation test showed a significantly positive correlation between wall motion scores and LGE scores (*r* = 0.488, *p* < 0.001).

**Table 2:** Distribution of myocardial segments in CTO territory according to scar formation.

	**0%**	**1–25%**	**26–50%**	**51–75%**	**>75%**	**Total**
**Distribution of the myocardial segments with different extent of myocardial scar**						
LAD (*n* = 71)	336 (68%)	34 (7%)	32 (6%)	49 (10%)	46 (9%)	497
LCX (*n* = 21)	63 (60%)	9 (9%)	15 (14%)	8 (8%)	10 (10%)	105
RCA (*n* = 102)	296 (58%)	51 (10%)	78 (15%)	44 (9%)	41 (8%)	510
Total	695 (63%)	94 (8%)	125 (11%)	101 (9%)	97 (9%)	1,112

*CTO, chronic total occlusion; LAD, left anterior descending; LCX, left circumflex; RCA, right coronary artery*.

**Figure 2:**
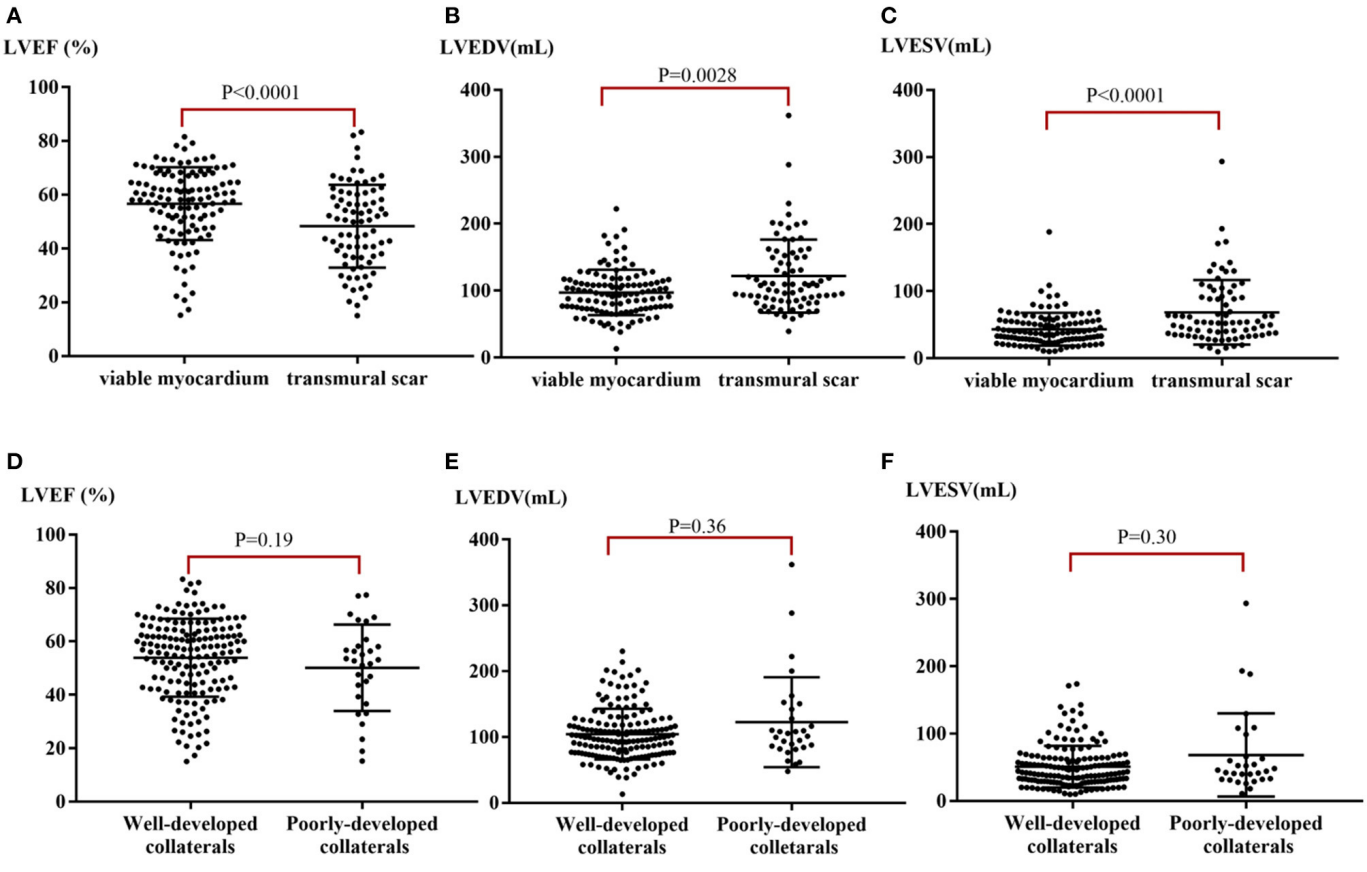
LV function stratified by myocardial viability and collaterals. **(A)** Median LVEF stratified by the extent of myocardial scar. **(B)** Median LVEDV stratified by the extent of myocardial scar. **(C)** Median LVESV stratified by the extent of myocardial scar. **(D)** Median LVEF stratified by well- or poorly-developed collaterals. **(E)** Median LVEDV stratified by well- or poorly-developed collaterals. **(F)** Median LVESV stratified by well- or poorly-developed collaterals. LVEF, left ventricular ejection fraction; LVEDV, left ventricular end-diastolic volume; LVESV, left ventricular end-systolic volume.

Left ventricular dysfunction was observed in 67 enrolled patients (35%) with an average LVEF of 36.4 ± 9.4%. Over 50% LGE was observed in 27% (103/381) of segments in CTO territory of patients with reduced LVEF (<50%), but only in 13% (95/731) of segments in CTO territory of those patients with normal systolic function (≥50%, *p* < 0.001). Patients with viable myocardia (LGE 0–50%) had higher LVEF (56.7 ± 13.5% vs. 48.3 ± 15.4%, *p* < 0.001, Figure 2A) and lower LV end-diastolic volume (LVEDV) (97.3 ± 33.8 ml vs. 121.7 ± 54.4 ml, *p* = 0.003, Figure 2B) and LV end-systolic volume (LVESV) (43.3 ± 24.4 ml vs. 68.6 ± 48.1 ml, *p* < 0.001, Figure 2C) than those with transmural scar.

### Myocardial Viability, LV Function, and Collateral Status

Poorly-developed angiographic collaterals were observed in 30 patients (15%), while well-developed collaterals were found in 164 patients (85%). Interobserver and intraobserver agreement for the Rentrop grading of collaterals in 50 (26%) randomly selected patients was high (intraobserver agreement = 90%; the Cohen's kappa = 0.62, 95% CI: 0.32–0.92).

**Figure 3:**
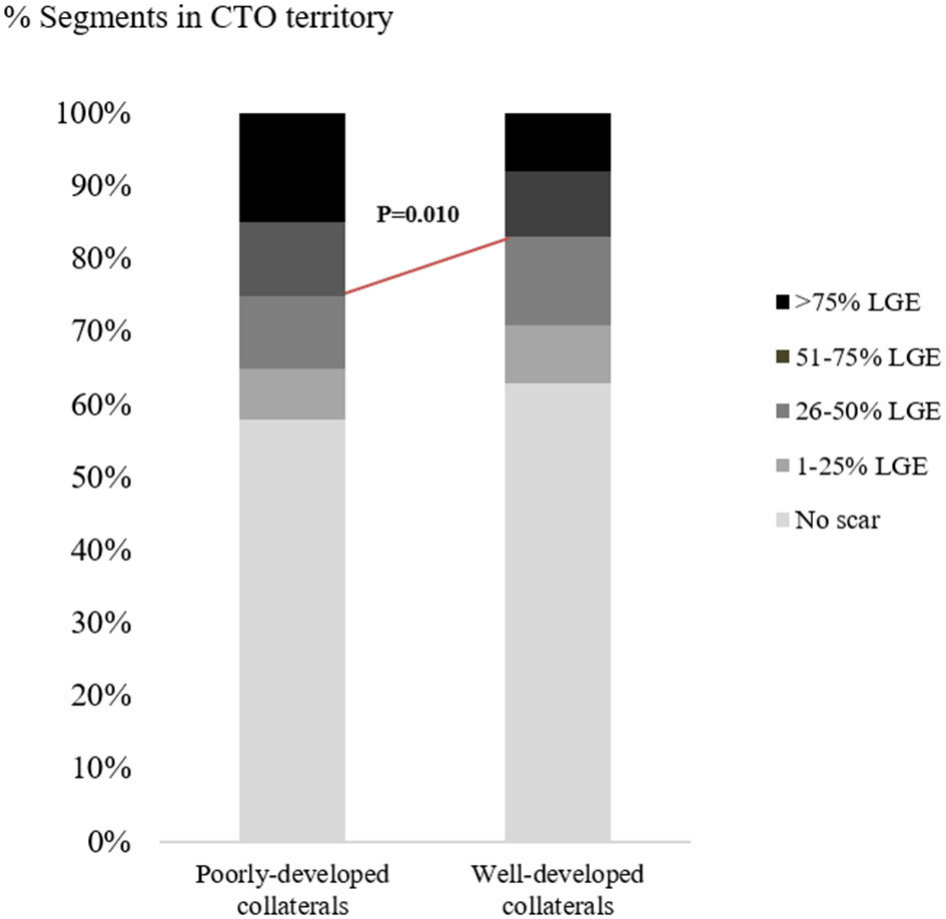
Distribution of myocardial segments subtended by CTO arteries according to scar formation. CTO, chronic total occlusion; LGE, late gadolinium enhancement.

Of the patients with poorly-developed collaterals, a total of 21 (70%) patients had a myocardial scar in CTO territory, while 65% of patients with well-developed collaterals (107/164) showed scar in CTO territory. There was no significant correlation between the presence of myocardial scar and collateral status (*p* = 0.680). Nevertheless, 25% of segments subtended to CTO arteries showed over 50% LGE in patients with poorly-developed collaterals, while only 17% of segments subtended to CTO arteries showed over 50% LGE in those patients with well-developed collaterals (*p* = 0.010; Figure 3). Additionally, more viable CTO-related segments were found in the presence of well-developed collaterals than in the presence of poorly-developed collaterals, despite the non-statistically significant difference [591 out of 934 (63%) vs. 104 out of 178 (58%) segments, *p* = 0.237].

**Figure 4:**
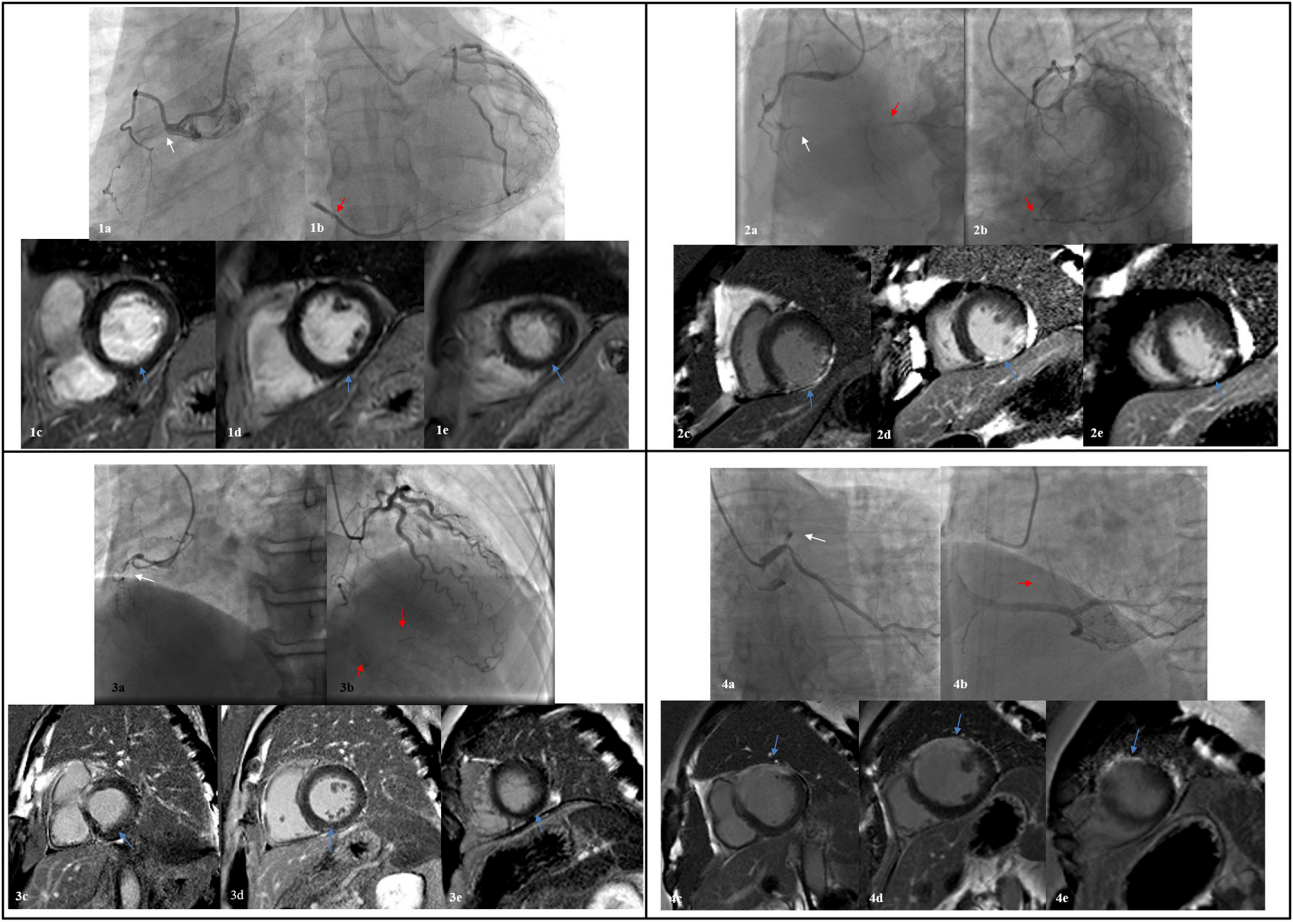
Four typical case scenarios displaying patients with well- or poorly-developed collaterals and different extent of myocardial scar. 1: a, CTO in proximal RCA (white arrow); b, a well-developed epicardial collateral from LCX (red arrow); c-e, no myocardial scar of the inferior wall was observed from basal, intermediate, and to apex level. 2: a, CTO in distal RCA (white arrow); b, well-developed collaterals from LCX and septum branches (red arrow); c-e, transmural scar of the inferior wall was observed from basal, intermediate, and to apex level. 3: a, CTO in proximal RCA (white arrow); b, poorly-developed collaterals from septum branches (red arrow); c-e, no myocardial scar of the inferior wall was observed from basal, intermediate, and to apex level. 4: a, CTO in proximal LAD (white arrow); b, poorly-developed collaterals from septum branches (red arrow); c-e, transmural myocardial scar of the anterior wall was observed from basal, intermediate, and to apex level. CTO, chronic total occlusion; RCA, right coronary artery; LCX, left circumflex; LAD, left anterior descending.

There were no significant differences between mean LVEF and ventricular volume in patients with poorly-developed collaterals when compared with those with well-developed collaterals (*p*-values: LVEF = 0.191, LVEDV = 0.360, LVESV = 0.300, Figures 2D–F). Typical case scenarios displaying patients with well- or poorly-developed collaterals and patients with different extents of myocardial scar are shown in Figure 4.

## Discussion

In this study, we assessed the myocardial viability and functional status in CTO territory by using CMR and investigated the correlation between the prevalence of myocardial viability, LV systolic function, and collateral status. The prevalence of myocardial scar in CTO territory was >50%, while only 18% of segments had transmural scar. Patients with transmural scar had worse systolic functioning. Although collateral status was not related to the presence of myocardial scar or to LV function, well-developed collaterals reduced the number of segments with transmural scar in CTO territory.

### Assessment of Myocardial Viability to Predict the Value of CTO PCI

We reported a higher prevalence of myocardial scar detected by CMR than traditional clinical evidence in this study. However, this finding was consistent with previous work (20). We attribute this to the high sensitivity of CMR for recognizing endocardial scar, while the occurrence of myocardial infarction symptoms or Q waves on ECG relates more to transmural necrosis. Despite the considerable prevalence of myocardial scar, less than one-fifth of the segments showed transmural scar, suggesting that severe ischemic injury is limited in CTO territory. However, since the benefits of revascularization for CTO are still controversial, many researchers pay more attention to factors that predict better outcomes following CTO PCI. Studies have shown that myocardial viability is an important outcome predictor, as confined endocardial scar or absolute viability has been associated with functional recovery (21, 22). Schumacher et al. previously reported that 76% of patients had evidence of LGE and that only 5% of CTO segments demonstrated transmural scar tissue that could be detected by CMR (23). The notion that the ischemic myocardial scar is common but most limited within the endocardium would be consistent with our results. Particularly, an evident extensive myocardial scar was observed beyond CTO territories in a few patients, despite adjustment according to the dominance of coronary artery. Indeed, the complexity of vascular variability made it difficult to assign the 17 segments of LV to specific coronary arteries. As previously reported (17), the most specific segments including anterior, anteroseptal segments correspond to LAD, but no segments can be exclusively attributed to RCA or LCX. Additionally, Yajima et al. (24) also reported myocardial scar in territories adjacent or remote to CTO territories in a chronic myocardial infarction (MI) adult porcine model and explained it by endothelium swelling and microvasculature disruption. Therefore, the variable vascular territories and extensive microvasculature injury could account for the bias of myocardial viability analysis.

### Functional Status and Recovery

Ischemia is estimated to be responsible for around two-thirds of heart failure cases (25). In patients with ischemic ventricular dysfunction, the presence of CTO is associated with higher morbidity and poor prognosis (26). In this study, we reported that 35% of patients had impaired systolic functioning and that patients with transmural scar had worse functional statuses. A few observational studies investigating the outcomes of CTO PCI among patients with LV dysfunction have shown substantial LVEF improvement and decreased cardiac mortality, especially in those with severely impaired systolic functions (27–30). Interventional procedures are more challenging in these complicated patients. A study demonstrated that PCI was a safe strategy in patients with low LVEF ( ≤ 35%), as the angiographic success rate was high and similar to that in patients with LVEF > 35% without more periprocedural complications (31). However, these outcomes are still limited in non-randomized controlled trials (RCTs). The REVASC trial (A Randomized Trial to Assess Regional Left Ventricular Function After Stent Implantation in Chronic Total Occlusion) did not show any benefits related to CTO PCI. Therefore, experts recommend both the viability and functional assessment in patients with ischemic cardiomyopathy for CTO PCI (32).

### Collaterals, Myocardial Viability, and Functional Status

There was no significant difference in the prevalence of myocardial scar or systolic functioning in patients with well- or poorly-developed collaterals and there were fewer non-viable segments in the CTO territory supplied by well-developed collaterals. The presence of well-developed collaterals may not directly predict myocardial viability, but could protect the myocardium from severe ischemic injury to some extent. In fact, whether collaterals could have protective effects on myocardial viability and contractility have long been a matter of debate. In a recent study, patients with well-developed collaterals showed less myocardial scar as assessed by quantitative CMR analysis and more retained systolic function in CTO territory (23). Previously, a few studies reported that collaterals had lower sensitivity for predicting myocardial viability (33, 34). The blood supply from collaterals to the totally occluded territory could be limited, especially when myocardial oxygen consumption increases. Additionally, myocardial viability and contractility could be affected by many concomitant factors including multiple-vessel stenosis and microvascular dysfunction. In the subgroup analysis of the EXPLORE (Evaluating Xience and Left Ventricular Function in Percutaneous Coronary Intervention on Occlusions After ST-Elevation Myocardial Infarction) trial, the presence of well-developed collaterals was shown to correlate with better outcomes. However, well-developed collaterals did not translate into better clinical outcomes. A recent meta-analysis showed that the presence of well-developed collaterals is not well-related to lower rates of acute MI (AMI) or mortality, but does increase the likelihood of successful CTO PCI (35, 36). These results indicated that well-developed collaterals should not be the only factor that affects prognosis.

### Limitations

There are several limitations inherent in this study. First, it was a single-center and observational study with a small sample size that enrolled patients with a range of LVEF—meaning the population with impaired LVEF accounted for a minority of patients. This does, however, reflect a real-world trend. Second, the LGE images were analyzed by using a semi-quantitative visual method rather than a quantitative method. However, the utility of quantitative CMR analysis by using commercial software is still limited and thresholds for signal intensity are not unified. Third, stress perfusion imaging was not performed on our patients, meaning that we lacked analysis of ischemic burden in CTO territory. Finally, collateral circulation was only assessed by using visualized angiography and functional intravascular evaluations were not employed.

## Conclusion

This study demonstrates that myocardial injuries in CTO territory are common, but those non-viable myocardia only account for a minority. Further, transmural myocardial scar appears to be associated with worse functional outcomes. Finally, well-developed collaterals are not related to the prevalence of myocardial scar or systolic functioning, but can reduce the number of segments with non-viable scar that are subtended by CTO arteries.

## Data Availability Statement

The raw data supporting the conclusions of this article will be made available by the authors, without undue reservation.

## Ethics Statement

The studies involving human participants were reviewed and approved by Beijing Anzhen Hospital. The patients/participants provided their written informed consent to participate in this study.

## Author Contributions

XY, JT, LZ, WD, HM, JianL, JiahL, YHa, HZ, JA, YHe, and XS: conceptualization. JT and JianL: data curation. XY and JT: formal analysis. JT and XS: funding acquisition. XY, JT, and JianL: investigation. LZ, WD, HM, HZ, YHe, and XS: methodology. XY, JT, JianL, and XS: project administration. XS: resources. LZ, WD, HM, and YHe: software. YHe and XS: supervision, validation, and visualization. XY and JT: writing—original draft. LZ, YHe, and XS: writing—review and editing. All authors contributed to the article and approved the submitted version.

## Funding

This work was supported by Capital Health Development Research Project (No. 2018-2-2063), National Natural Science Foundation of China (Nos. 81971569, 81670324, and 81671650), Beijing Lab for Cardiovascular Precision Medicine (PXM2018_014226_000013), Beijing Municipal Science and Technology Project (Z161100000516139), 2018 Beijing Excellent Talent Fund (NO. 2018000021469G241).

## Conflict of Interest

JA was employed by the company Siemens Shenzhen Magnetic Resonance Ltd. The remaining authors declare that the research was conducted in the absence of any commercial or financial relationships that could be construed as a potential conflict of interest.

## Publisher's Note

All claims expressed in this article are solely those of the authors and do not necessarily represent those of their affiliated organizations, or those of the publisher, the editors and the reviewers. Any product that may be evaluated in this article, or claim that may be made by its manufacturer, is not guaranteed or endorsed by the publisher.
