# Corrigendum: Myocardial Viability, Functional Status, and Collaterals of Patients With Chronically Occluded Coronary Arteries

**DOI:** 10.3389/fcvm.2022.877972

**Published:** 2022-03-18

**Authors:** Xueyao Yang, Jinfan Tian, Lijun Zhang, Wei Dong, Hongzhi Mi, Jianan Li, Jiahui Li, Ye Han, Huijuan Zuo, Jing An, Yi He, Xiantao Song

**Affiliations:** ^1^Department of Cardiology, Beijing Anzhen Hospital, Capital Medical University, Beijing, China; ^2^Department of Radiology, Beijing Anzhen Hospital, Capital Medical University, Beijing, China; ^3^Department of Nuclear Medicine, Beijing Anzhen Hospital, Capital Medical University, Beijing, China; ^4^Department of Radiology, Beijing Friendship Hospital, Capital Medical University, Beijing, China; ^5^Department of Community Health Research, Beijing Institute of Heart, Lung and Blood Vessel Disease, Beijing Anzhen Hospital, Capital Medical University, Beijing, China; ^6^Siemens Shenzhen Magnetic Resonance Ltd., Shenzhen, China

**Keywords:** chronic total occlusion, myocardial viability, coronary artery disease, cardiovascular magnetic resonance, cardiac function

In the **Abstract section** of original article, there was an error. There is a typo in the “Conclusion” of the abstract of this paper, where “Myocardial infarction detected by CMR is widespread among patients with CMO” should be changed to “Myocardial infarction detected by CMR is widespread among patients with CTO.”

In the original article, there were some mistakes in Table 1 as published. The total number of patients who had hypertension should be 115 (59%) instead of 125 (64%). The mean ± standard deviation of LVEF (%) of all patients, patients with no scar in CTO territory and patients with scar in CTO territory should be 53.3 ± 14.9, 58.3 ± 12.7, 50.7 ± 15.2, respectively. The mean ± standard deviation of LVEDV (mL) of all patients, patients with no scar in CTO territory and patients with scar in CTO territory should be 107.1 ± 44.8, 90.0 ± 32.4, and 115.9 ± 47.8, respectively. The mean ± standard deviation of LVESV (mL) of all patients, patients with no scar in CTO territory and patients with scar in CTO territory should be 53.3 ± 37.8, 38.7 ± 20.1, and 61.1 ± 42.4, respectively. The corrected Table 1 appears below.

**Table 1:** Baseline characteristics.

	**All patients (*n* = 194)**	**No scar in CTO territory on CMR (*n* = 66)**	**Scar detected in CTO territory by CMR (*n* = 128)**	***P***
Age	57 ± 10	56 ± 10	58 ± 10	0.067
Male	160 (82%)	46 (70%)	114 (89%)	<0.0001
Smoking	102 (53%)	25 (38%)	77 (60%)	0.004
Hypertension	115 (59%)	29 (44%)	86 (67%)	0.002
Diabetes	60 (31%)	21 (32%)	39 (30%)	0.871
Hyperlipemia	80 (41%)	28 (42%)	52 (41%)	0.878
Prior PCI	52 (27%)	11 (17%)	37 (29%)	0.079
Previous MI	45 (23%)	9 (14%)	36 (28%)	0.031
Q wave	22 (11%)	1 (2%)	21 (16%)	0.001
**Angiographic presentation**				
CTO location				
LAD	71 (37%)	31 (46%)	40 (31%)	–
LCX	21 (11%)	6 (12%)	15 (12%)	–
RCA	102 (53%)	29 (42%)	73 (57%)	–
Successful CTO revascularization	126 (65%)	49 (71%)	77 (60%)	0.058
Concomitant non-CTO lesion	79 (41%)	28 (38%)	51 (40%)	0.759
**CMR characteristics**				
Scar on CMR in CTO territory	128 (66%)	–	–	–
Wall motion abnormality in CTO territory	101 (52%)	13 (20%)	88 (75%)	<0.0001
LVEF (%)	53.3 ± 14.9	58.3 ± 12.7	50.7 ± 15.2	0.001
LVEDV (mL)	107.1 ± 44.8	90.0 ± 32.4	115.9 ± 47.8	<0.0001
LVESV (mL)	53.5 ± 37.8	38.7 ± 20.1	61.1 ± 42.4	<0.0001

*CTO, chronic total occlusion; CMR, cardiovascular magnetic resonance imaging; LAD, left anterior descending coronary artery; LCX, left circumflex coronary artery; LVEF, left ventricular ejection fraction; LVESV, left ventricular end-systolic volume; LVEDV, left ventricular end-diastolic volume; PCI, percutaneous coronary intervention; RCA, right coronary artery*.

The authors apologize for this error and state that this does not change the scientific conclusions of the article in any way. The original article has been updated.

## Publisher's Note

All claims expressed in this article are solely those of the authors and do not necessarily represent those of their affiliated organizations, or those of the publisher, the editors and the reviewers. Any product that may be evaluated in this article, or claim that may be made by its manufacturer, is not guaranteed or endorsed by the publisher.

